# An Efficient Method for Solving Router Placement Problem in Wireless Mesh Networks Using Multi-Verse Optimizer Algorithm

**DOI:** 10.3390/s22155494

**Published:** 2022-07-23

**Authors:** Le Huu Binh, Tung Khac Truong

**Affiliations:** 1 Faculty of Information Technology, University of Sciences, Hue University, Hue City 49000, Vietnam; lhbinh@hueuni.edu.vn; 2 Faculty of Information Technology, School of Engineering and Technology, Van Lang University, Ho Chi Minh City 70000, Vietnam

**Keywords:** mesh router placement, multi-verse optimizer algorithm, wireless mesh network, network design

## Abstract

Wireless Mesh Networks (WMNs) are increasingly being used in a variety of applications. To fully utilize the network resources of WMNs, it is critical to design a topology that provides the best client coverage and network connectivity. This issue is solved by determining the best solution for the mesh router placement problem in WMN (MRP-WMN). Because the MRP-WMN is known to be NP-hard, it is typically solved using approximation algorithms. This is also why we are conducting this work. We present an efficient method for solving the MRP-WMN using the Multi-Verse Optimizer algorithm (MVO). A new objective function for the MRP-WMN is also proposed, which takes into account two important performance metrics, connected client ratio and connected router ratio. Experiment results show that when the MVO algorithm is applied to the MRP-WMN problem, the connected client ratio increases by 15.1%, 11.5%, and 5.9% on average, and the path loss reduces by 1.3, 0.9, and 0.6 dB when compared to the Genetic Algorithm (GA), Particle Swarm Optimization (PSO), and Whale Optimization Algorithm (WOA), respectively.

## 1. Introduction

**Figure 1:**
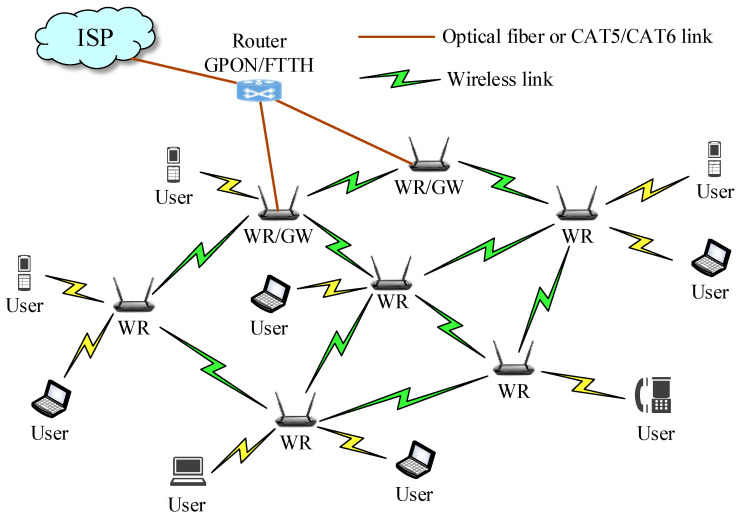
The general architecture of the WMN [21].

Wireless communication is now one of the most common solutions in network technology. At the access layer, WMNs are commonly used in the local area networks of government agencies, businesses, schools, and hospitals, etc. [1,2]. Figure 1 depicts the general architecture of the WMN, in which nodes are connected via a wireless transmission channel to form a mesh topology. Mesh client (MC), mesh router (MR), and mesh router with gateway (MR/GW) are the three types of nodes in the WMN. To access the internet, the MCs connect to the MRs or MR/GWs via a wireless communication medium.

To improve the performance of WMNs, it is essential to study architectures, models, and network topologies. Many research groups have recently completed this [3,4,5,6,7,8], where the MRP-WMN has become an important topic. Because this problem is known as NP-hard [9], conventional algorithms cannot solve it. As a result, the MRP-WMN problem can only be solved by approximation optimization algorithms, such as heuristic and meta-heuristic [3,4,10,11]. The majority of published papers consider the MRP-WMN problem in two ways, stationary mesh router placement [10,12,13,14] and dynamic mesh router placement based on client mobility [15,16,17,18]. The Coyote Optimization Algorithm (COA) was used by the authors of [3] to solve the MRP-WMN problem. Their proposed algorithm simultaneously optimized two important performance metrics, network connectivity and user coverage. The authors demonstrated that the proposed algorithm outperforms other well-known optimization algorithms using a Matlab simulation method. Although the work in [3] has significantly improved network connectivity and user coverage metrics, this work has not yet considered connectivity to the gateway of mesh routers. This is a requirement for covered users to access the internet. As a result, the proposed algorithm in [3] is only appropriate for WMN peer-to-peer network models, such as mobile ad hoc network (MANET), which are only concerned with data transmission between users. The authors of [10] used the algorithm accelerated PSO algorithm (APSO) to solve MRP-WMN. The APSO algorithm was chosen for this project because of its fast convergence and low computational complexity. In terms of coverage and connectivity, simulation results using the C and MATLAB programming languages show that the APSO algorithm outperforms the linearly decreasing weight particle swarm optimizer (LDWPSO) algorithm [19]. By optimizing the metrics of the coverage and connectivity, the authors of [11] have proposed an optimal algorithm, namely the Chemical Reaction Optimization algorithm (CRO), to solve the MRP-WMN. The CRO algorithm is inspired by the interactions between molecules in chemical reactions to reach a low, stable energy state. Simulation results show that their proposed algorithm can improve client coverage and network connectivity compared to the GA algorithm. In [20], the authors have proposed a Genetic Algorithm in addition to the approach that was derived from the circle packing problem. Their proposed algorithm has two goals, maximizing network connectivity as well as coverage area. The testing findings demonstrated the effectiveness of their approach in generating high-quality and appropriate mesh router node placement solutions in WMN.

Another study utilized a genetic algorithm and simulated annealing to find a low-cost WMN configuration while satisfying constraints and figuring out how many gateways were needed [22]. The performance of the genetic algorithm and simulated annealing in decreasing WMN network expenses while maintaining quality of service (QoS) was demonstrated in experiments. The new models are shown to outperform existing solutions significantly. In [23], the QoT was also considered in the MPR-WMN problem. The authors have presented a novel particle swarm optimization approach to maximize both network connectivity and client coverage. The QoS constraints of this work are delay, relay load, and Internet gateway capacity.

We concluded from our review of the above publications that approximation optimization methods can be employed effectively to solve the MRP-WMN problem. We continue to develop this research topic in this paper. Using the Multi-Verse Optimizer algorithm (MVO), we provide an efficient technique for solving the MRP-WMN problem. The following are the primary contributions of this paper:

- Proposed an efficient method for solving the RNP-WMN problem using an MVO algorithm to improve the percentage of covered clients under the connection constraint to the gateway.
- Formulate a multi-objective function for the RNP-WMN problem to simultaneously maximize two important performance metrics, namely connected client ratio and connected router ratio.
- Evaluation and comparison of the performance of the MVO algorithm with algorithms PSO, WOA and GA in solving RNP-WMN problem.

The next sections of this paper are organized as follows. Section 2 describes the formulation of the RNP-WMN problem. Section 3 presents the MVO algorithm and its application to solve the RNP-WMN problem. Section 4 presents the simulation results and discussion. Finally, concluding remarks and promising future study items are given in Section 5.

## 2. RNP-WMN Problem

To provide clarity in formulating the RNP-WMN problem, we first list the important notation used for the problem formulation, which is shown in Table 1.

### 2.1. System Model

Mathematically, a WMN can be represented as an undirected graph with the formula G=(V,E), where *V* represents the set of network nodes and *E* represents the set of wireless links connecting these nodes. The WMN has three different types of nodes: mesh routers, mesh clients, and gateway routers. The following is a representation of these nodes:

- $R=\{r_{1},r_{2},\ldots ,r_{m}\}$ the set of mesh routers. The coverage radius of each mesh router is a $d_{CR}$ meter. Two mesh routers $r_{i}$ and $r_{j}$ can be connected by a wireless link if and only if the distance between them is less than or equal to twice the coverage radius. i.e., $d\left(r_{i},r_{j}\right)\leq 2d_{CR}$, where $d(r_{i},r_{j})$ is the distance between routers $r_{i}$ and $r_{j}$, determined by
  (1) $$d\left(r_{i},r_{j}\right)=\sqrt{{\left(x_{r_{i}}-x_{r_{j}}\right)}^{2}+{\left(y_{r_{i}}-y_{r_{j}}\right)}^{2}}$$
  with pairs $\left(x_{r_{i}},y_{r_{i}}\right)$ and $\left(x_{r_{j}},y_{r_{j}}\right)$ are the coordinates of the mesh routers $r_{i}$ and $r_{j}$, respectively.
- $C=\{c_{1},c_{2},\ldots ,c_{n}\}$ is the set of mesh clients. If the client $c_{i}$ is within the coverage area of the router $r_{i}$ (i.e., $d\left(c_{i},r_{i}\right)\leq d_{CR}$), a wireless link exists between $c_{i}$ and $r_{i}$. In case a client is within the coverage area of many routers, it will connect to the nearest router
- $GW=\{r_{gw_{1}},r_{gw_{2}},\ldots ,r_{gw_{k}}\}$ is the set of gateway routers. In real network models, the mesh routers can connect to the gateway routers by a wired or wireless transmission medium. In the context of this work, the wireless transmission medium is used to connect them. If the mesh router $r_{i}$ is in the coverage area of the gateway router $r_{gw_{j}}$, there is a wireless link that connects $r_{i}$ and $r_{gw_{j}}$. In this case, the mesh router $r_{i}$ acts as a mesh router with a gateway (as we describe the principle of WMN in Figure 1).

### 2.2. Problem Formulation

Consider a WMN in a 2D area of dimensions W×H, where the number of mesh routers, mesh clients and gateway routers are *m*, *n* and *k*, respectively. The RNP-WMN problem is stated as finding the optimal set of locations for m mesh routers (set $\left\{\left(x_{r_{i}},y_{r_{i}}\right)|\forall r_{i}\in R\right\}$, as defined in Table 1), depending on the given set of locations of mesh clients. To formulate this problem, we first define some concepts.

#### 2.2.1. Connected Router

The mesh router $r_{i}$ is a connected router if and only if there exists a path from it to at least one gateway router in the WMN.

**Figure 2:**
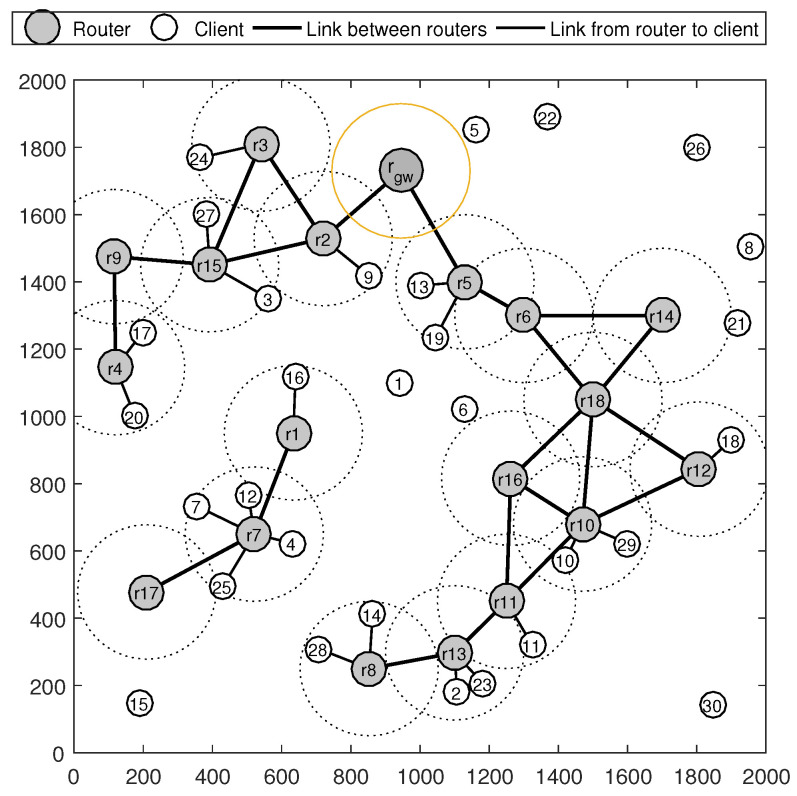
An example of the topology of the WMN.

To better understand the concepts of connected routers, consider the example shown in Figure 2, where a WMN consists of 18 mesh routers, 30 mesh clients and 1 gateway router ($r_{gw}$), located in an area of 2000 × 2000 meters. For the current state, mesh routers $r_{2},r_{3},r_{4},r_{5},r_{6},r_{8},r_{9},r_{10},r_{11},r_{12},r_{13},r_{14},r_{15},r_{16}$ and $r_{18}$ are connected routers because there are paths from these mesh routers to the gateway router $\left(r_{gw}\right)$. The mesh routers $r_{1}$, $r_{7}$ and $r_{17}$ are not connected routers because there is no path from these routers to the gateway router.

#### 2.2.2. Connected Router Ratio

The connected router ratio is defined as the ratio of the number of connected routers to the number of routers in a WMN, calculated by

(2) $$\Psi \left(G\right)\;=\frac{\sum_{i=1}^{m}\alpha_{r_{i}}}{m}\times 100\left(\%\right)$$

where *m* is the number of mesh routers, $\alpha_{r_{i}}$ is a variable that indicates whether router $r_{i}$ is a connected router or not, defined as follows

(3) $$\alpha_{r_{i}}=\left\{\begin{array}{cc} 1 & \mathrm{if}r_{i}\mathrm{is}a\mathrm{connected}\mathrm{router}\\ 0 & otherwise \end{array}\right.$$

#### 2.2.3. Connected Client

The mesh client $c_{i}$ is a connected client if and only if it is covered by at least one connected router.

Going back to the example in Figure 2, we can observe that client $c_{9}$ is a connected client because it is covered by the connected router $r_{2}$. The client $c_{20}$ is also a connected client because it is covered by the connected router $r_{4}$. However, clients $c_{1}$, $c_{5}$, $c_{6}$, $c_{8}$, $c_{15}$, $c_{21}$, $c_{22}$, $c_{26}$ and $c_{30}$ are not connected clients because they are not covered by any connected router. Although clients $c_{4}$, $c_{7}$, $c_{12}$, $c_{16}$ and $c_{25}$ are covered by routers $r_{1}$ and $r_{7}$, these clients are also not connected clients because routers $r_{1}$ and $r_{7}$ are not connected routers.

#### 2.2.4. Connected Client Ratio

The connected client ratio is defined as the ratio of the number of connected clients to the number of clients in a WMN, calculated by

(4) $$\Gamma \left(G\right)\;=\frac{\sum_{i=1}^{n}\beta_{c_{i}}}{n}\times 100\left(\%\right)$$

where *n* is the number of mesh clients, $\beta_{c_{i}}$ is a variable that indicates whether client $c_{i}$ is a connected client or not, defined as follows

(5) $$\beta_{c_{i}}=\left\{\begin{array}{cc} 1 & \mathrm{if}c_{i}\mathrm{is}a\mathrm{connected}\mathrm{client}\\ 0 & otherwise \end{array}\right.$$

The main goal of the RNP-WMN problem is to find the set of locations for *m* mesh routers (set $\left\{\left(x_{r_{i}},y_{r_{i}}\right)|\forall r_{i}\in R\right\}$) so that the network performance is the best. In this work, we focus on optimizing two important performance metrics, namely connected router ratio (Ψ(G)) and connected client ratio (Γ(G)), as defined in Section 2.2.2 and Section 2.2.4, respectively. Since the larger Ψ(G) and Γ(G) metrics, the better the network performance, the RNP-WMN problem is formulated as follows:

(6) $$\left\{\begin{array}{c} Maximize\;\Psi (G)\\ Maximize\;\Gamma (G) \end{array}\right.$$

subject to the following constraints:

(7) $$\begin{array}{cc} \; & 0<x_{r_{i}}<W \end{array}$$

(8) $$\begin{array}{cc} \; & 0<y_{r_{i}}<H \end{array}$$

where *W* and *H* are the width and the height of the WMN area, respectively.

The RNP-WMN problem as defined in (6) can be solved by optimization algorithms. In this work, we apply the Multi-Verse Optimizer Algorithm (MVO) to solve it, and more information is provided in the following sections.

## 3. MVO Algorithm and Its Application to Solve the RNP-WMN Problem

### 3.1. MVO Algorithm

Mirjalili et al. proposed the Multi-Verse Optimizer Algorithm (MVO), a nature-inspired algorithm [24]. The MVO method is founded on three cosmological ideas: white holes, black holes, and worm holes. It employs exploration, exploitation, and local search to find the best answer among a large number of candidate solutions.

The multi-verse theory, which emerged after the Big Bang theory, inspired MVO. The Big Bang theory states that there was a massive explosion that resulted in the existence of the universe we live in. According to the multi-verse theory, there were multiple big bangs, each of which resulted in the existence of a different universe. As a result, multi-verse believes that there are other universes besides the one we live in. Furthermore, according to the multi-verse theory, these universes can interact and collide with one another, and each universe has its own set of properties.

MVO was primarily inspired by three concepts from multi-verse theory: white holes, black holes, and wormholes. White holes have never been observed, but physicists believe they can be explained by the big bang or collisions between parallel universes. Black holes attract everything due to their strong gravitational pull. Finally, wormholes connect all parts of the universe and serve as a time/space tunnel through which objects can travel. The original MVO paper mentioned that every universe has an inflation rate that causes space to expand.

MVO is a population-based algorithm that searches in two stages: exploration and exploitation. The MVO algorithm considers solutions to be universes and variables within a solution to be variables within a universe, where *d* represents the number of objects, *n* represents the number of universes, $X_{j}^{best}$ is the *j*-th parameter of the best universe formed so far, the TDR factor is calculated by Equation (10), the WEP factor is calculated by Equation (9), $lb_{j}$ shows the lower bound of the *j*-th variable, $ub_{j}$ is the upper bound of the *j*-th variable, $x_{i}^{j}$ indicates the *j*-th parameter of the *i*-th universe, and $rand_{2},rand_{3},rand_{4}$ are random numbers in [0, 1]. Equations (11) and (12) are used to update the universes. In addition, each universe is given an inflation rate ($U_{i}$) based on the fitness function value for that universe, $NI(U_{i})$ is the normalized inflation rate of the ith universe. The MVO algorithm is demonstrated in Algorithm 1.

(9) $$WEP=min+t\times (\frac{max-min}{Max\_iter})$$

(10) $$TDR=1-{\left(\frac{t}{Max\_iter}\right)}^{\frac{1}{p}}$$

(11) $$x_{i}^{j}\left(t+1\right)=\{\begin{array}{cc} x_{k}^{j}\left(t\right), & rand_{1}<NI\left(U_{i}\right)\\ x_{i}^{j}\left(t\right), & rand_{1}\geq NI\left(U_{i}\right) \end{array}$$

(12) $$x_{i}^{j}=\{\begin{array}{cc} X_{j}^{best}+TDR\times \left(\left(ub_{j}-lb_{j}\right)\times rand_{4}+lb_{j}\right), & rand_{3}<0.5\\ X_{j}^{best}-TDR\times \left(\left(ub_{j}-lb_{j}\right)\times rand_{4}+lb_{j}\right), & otherwise \end{array}$$

**Algorithm 1:** The pseudo-code of the MVO algorithm

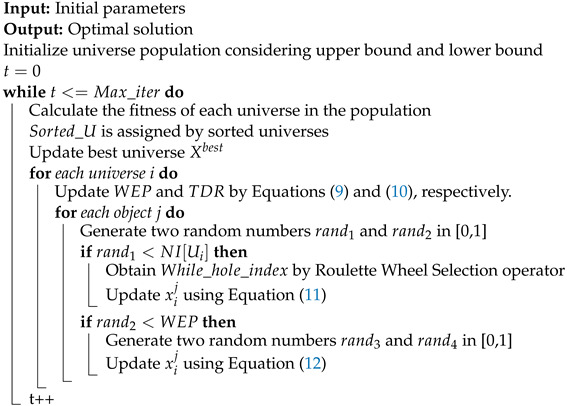



### 3.2. Application of the MVO Algorithm to Solve the RNP-WMN Problem

#### 3.2.1. Solution Presentation

**Figure 3:**
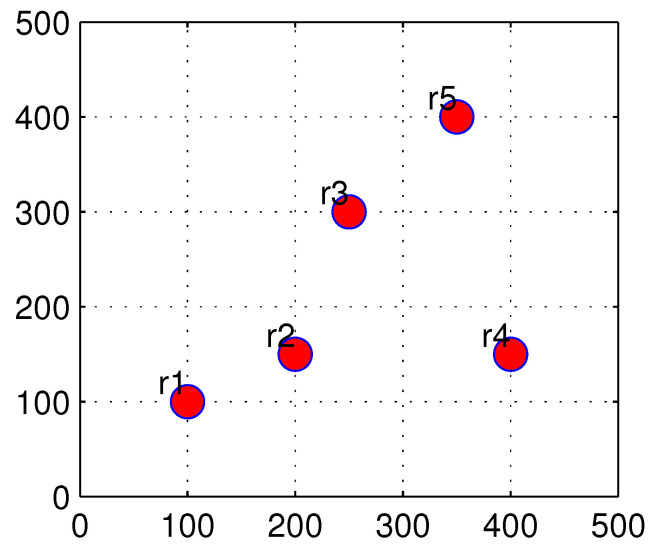
An example of the solution presentation.

Each solution to the mesh routers placement problem in WMN is a set of m coordinates corresponding to *m* locations to place *m* routers. In this paper, we use an array $X=\{x_{1},y_{1},x_{2},y_{2},\ldots ,x_{m},y_{m}\}$ to represent the found solution, where the pair $\left(x_{i},y_{i}\right)$ is the locations of the mesh router $r_{i}$. For example, consider a solution obtained as in (13):

(13) X={100,100,200,150,250,300,400,150,350,400},

This solution is the placement positions of five routers $r_{1}$, $r_{2}$, $r_{3}$, $r_{4}$ and $r_{5}$ at coordinates (100, 100), (200, 150), (250, 300), (400, 150) and (350, 400), respectively. The locations of these routers are illustrated in Figure 3.

#### 3.2.2. Objective Function

In this work, we focus on optimizing two important performance metrics, namely CRR and CCR, as defined in Section 2.2.2 and Section 2.2.4, respectively. To solve two maximize objectives functions, this research combines two maximize objectives functions to minimize a single objective function by the following formula:

(14) f(X)=1−(λΨ(G)+(1−λ)Γ(G))

where λ is a coefficient in the range [0, 1], which is used to control the optimal degree of metrics.

## 4. Performance Evaluation by Simulation

### 4.1. Simulation Scenarios

The performance of the MVO algorithm in solving the mesh router nodes placement problem is evaluated by a simulation using MATLAB. The setup of the MVO algorithm to solve this problem is presented in Table 2. The MVO algorithm is compared with the algorithms of GA [25], WOA [26] and PSO [27] in terms of user coverage, router connectivity, objective function value and coverage intensity. All experiments are performed on a Core i7 CPU 3.3 Ghz-CPU machine. Table 3 and Table 4 present the simulation assumptions, with Table 3 presenting the individual parameters for each algorithm and Table 4 presenting the common parameters for all four algorithms. We set up many different scenarios for cases where the number of mesh clients and mesh routers is different. Mesh clients are randomly distributed in the simulation area. In addition, there is a gateway router placed at a given location. This is the router of the internet service provider, and it acts as the gateway for the mesh client to access the internet. To ensure the same comparison condition, the position set of mesh clients and gateway router is the same for all algorithms. Each simulation scenario is run 30 times, and we use the average results of all times presented in this section.

**Figure 4:**
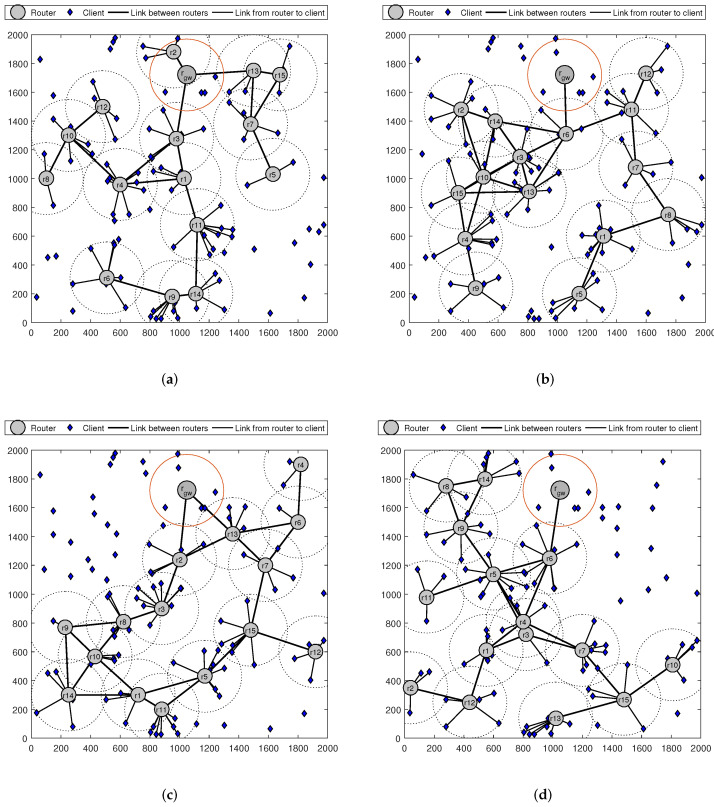
The network topologies of WMN obtained using algorithms MVO, WOA, GA and PSO. (**a**) GA. (**b**) PSO. (**c**) WOA. (**d**) MVO.

The results presented in Figure 4 are examples of the WMN topologies obtained by applying the GA, PSO, WOA and MVO algorithms for the mesh router’s node placement. In these cases, the number of mesh routers and mesh clients is 15 and 100, respectively. The circles with the center of a mesh router represent the coverage of that mesh router. The solid lines between two mesh routers or a mesh router and a mesh client indicate that these routers and clients are within the transmission area of each other. The results in Figure 4 have shown that the obtained network topology can be different depending on the optimal algorithm applied to place the mesh routers.

### 4.2. Performance Metrics and Network Instances

In our simulation models, the metrics of the connected client ratio (CCR), path loss and objective function value are used to evaluate and analyze the performance of GA, WOA, PSO and MVO algorithms in solving the mesh router node placement problem. The CCR is determined according to (4). Path loss (PL) is the signal power loss over the transmission medium. In the context of this paper, the free space transmission medium is considered for WMN, and the PL is defined as follows [28]:

(15) $$PL\;\left(dB\right)=10log_{10}\left[{\left(\frac{4\pi f_{c}d}{c}\right)}^{2}\right]$$

where $f_{c}$ is the carrier frequency, *c* is the speed of light ($≃3\times 10^{8}m/s$) and *d* is the distance between the transmitter and the receiver. In this paper, we focus on analyzing the PL between the mesh client and the nearest mesh router.

These performance metrics are evaluated through eight network instances (INS-1 to INS-8), as described in Table 5. The network instances of INS-1 to INS-2, INS-3 to INS-4 and INS-6 to INS-8 are used to evaluate the effect of the number of mesh routers, the number of mesh clients and the coverage radius of the mesh routers on network performance, respectively.

### 4.3. Impact of the Number of Mesh Routers

**Figure 5:**
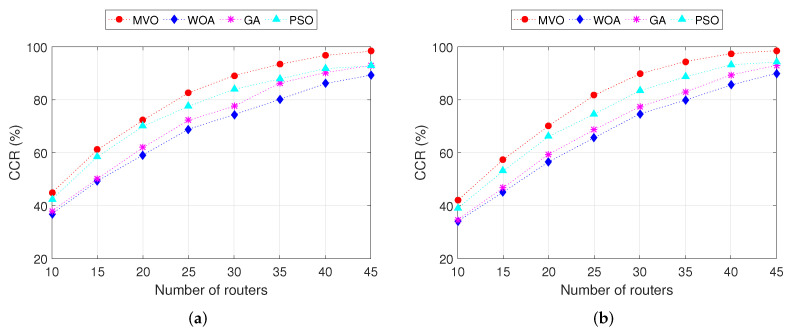
Performance comparison of the CCR versus the number of routers. (**a**) INS-1. (**b**) INS-2.

The results obtained in Figure 5 show the effect of the number of mesh routers on CCR, where we plot the CCR as a function of the number of the mesh routers. These results are obtained when executing INS-1 and INS-2. We can observe that the higher the number of mesh routers, the higher the CCR for all algorithms, where the CDR is the best for the case of the MVO algorithm. For example, consider the INS-1 (Figure 5a) with 30 mesh routers; the CCRs when using algorithms MVO, WOA, GA and PSO are 89.1%, 74.4%, 77.5% and 83.9%, respectively. Thus, the CCR of algorithm MVO is greater than that of algorithms WOA, GA and PSO by 14.7%, 11.6% and 5.2%, respectively. For the INS-2 (Figure 5a), the CCR is smaller than INS-1 due to the larger number of clients (350 clients). However, algorithm MVO always yields higher CCR than other algorithms. The details of the CCR values when executing INS-1 and INS-2 are shown in Table 6.

**Figure 6:**
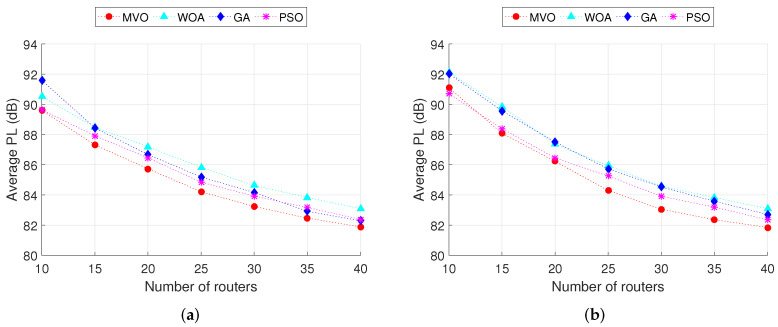
Performance comparison of the path loss versus the number of routers. (**a**) INS-1. (**b**) INS-2.

Next, we analyze the PL in WMN. As described in the previous section, PL is the signal power loss between the mesh client and the nearest mesh router, calculated according to (15). This is an important performance measure that greatly affects the quality of transmission (QoT) in WMN. The smaller the PL, the better the QoT. The results obtained in Figure 6 show the average PL in the entire network when executing INS-1 and INS-2 simulations. We can observe that the higher the number of mesh routers, the smaller the average PL for all algorithms, where the average PL is the best for the case of the MVO algorithm. This is because the larger the number of mesh routers, the higher the CCR (as analyzed in Figure 5), leading to a decrease in the average distance from the mesh client to the nearest mesh router. As a result, the average PL decreases. Comparing INS-1 (Figure 6a) and INS-2 (Figure 6a), we can observe that INS-1 performs better than INS-2 in terms of average PL. In other words, when the number of mesh clients is moderate (150 for this case), the QoT in the whole network is better than that of the case where the number of mesh clients is heavy (350 for this case). Comparing between four algorithms, MVO algorithm always gives the best QoT, and the difference in average PLs compared to algorithms WOA, GA and PSO are about 1.3, 0.9 and 0.6 dB, respectively.

**Figure 7:**
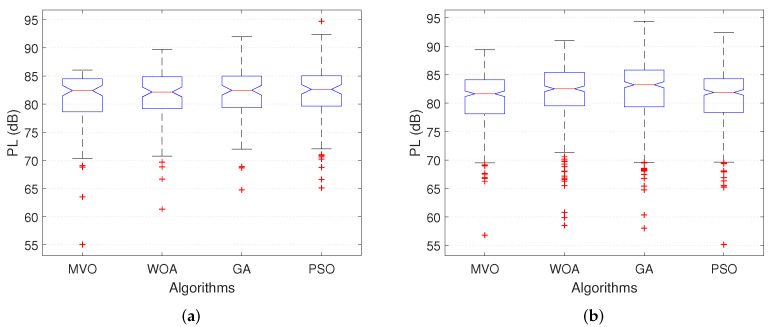
PL comparison of the algorithms MVO, WOA, GA and PSO. (**a**) INS-1 with 40 mesh routers. (**b**) INS-2 with 40 mesh routers.

The box plots in Figure 7 show the distribution of PL values for all clients. These results are obtained through INS-1 and INS-2 simulations with 40 mesh routers. We can observe that the MVO algorithm gives better PL than other algorithms for both INS-1 and INS-2. Considering the results of INS-1 (Figure 7a), the PL of the MVO algorithm ranges from 70.4 to 86.1 dB. In addition, there are four outliers of 55.1, 63.5, 68.8 and 69.1 dB. Meanwhile, the PL of algorithms WOA, GA and PSO range from 70.8 to 89.7 dB, 72 to 92 dB and 72 to 94.7 dB, respectively. There are also some outliers between 60.5 and 71 dB. Thus, the PL of the MVO algorithm is better than that of the WOA, GA and PSO algorithms.

### 4.4. Impact of the Number of Mesh Clients

**Figure 8:**
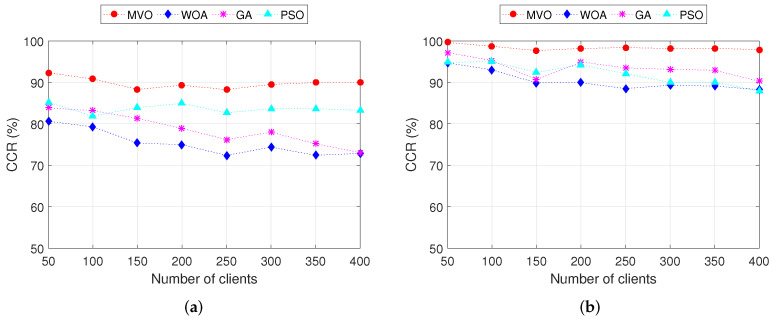
Performance comparison of the CCR versus the number of clients. (**a**) INS-3. (**b**) INS-4.

In this section, we analyze the effect of the number of mesh clients on the performance of the mesh routers placement algorithms in WMN. The charts in Figure 8 show the CCR when executing INS-3 and INS-4 with 30 and 45 mesh routers, respectively. The number of mesh clients is varied from 50 to 400. We can observe that for GA and PSO algorithms, the CCR decreases with the increase in the number of mesh clients. For MVO and WOA algorithms, as the number of mesh clients increases, the CCR changes only slightly. Comparing four algorithms, the MVO gives the best CCR for both INS-3 and INS-4. Consider an example in INS-3 (Figure 8a) with 300 mesh clients, the CCRs when using algorithms MVO, WOA, GA and PSO are 89.5%, 74.4%, 78.0% and 83.6%, respectively. Thus, the CCR of algorithm MVO is greater than that of algorithms WOA, GA and PSO by 15.1%, 11.5% and 5.9%, respectively. For the INS-4 (Figure 8b), the CCR is greater than INS-3 due to the larger number of mesh routers (45 routers for this instance). Specifically, algorithm MVO yields higher CCR than other algorithms. The details of the CCR values when executing INS-3 and INS-4 are shown in Table 7.

**Figure 9:**
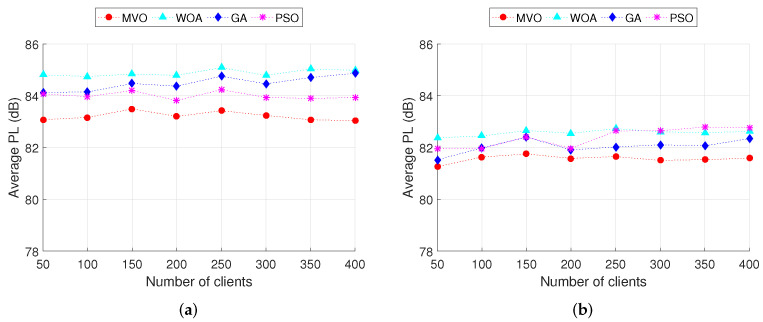
Performance comparison of the path loss versus the number of clients. (**a**) INS-3. (**b**) INS-4.

For the impact of the number of mesh clients on the PL, the simulation results are shown in Figure 9, where we plot the average PL as a function of the number of mesh clients. These results are obtained when executing INS-3 and INS-4. We can observe that the average PL does not change much according to the change in the number of mesh clients for both INS-3 and INS-4. Comparing INS-3 (Figure 9a) and INS-4 (Figure 9b), the average PL of INS-4 is better than that of INS-3. This is because the number of mesh routers of INS-4 is larger than that of INS-3 (40 routers for INS-4 and 30 routers for INS-3), resulting in the CCR of INS-4 being larger than that of INS-3. As a result, INS-4 outperforms INS-3 in terms of average PL. Among the algorithms MVO, WOA, GA and PSO, MVO gives the highest efficiency.

### 4.5. Impact of Coverage Radius of the Mesh Routers

**Figure 10:**
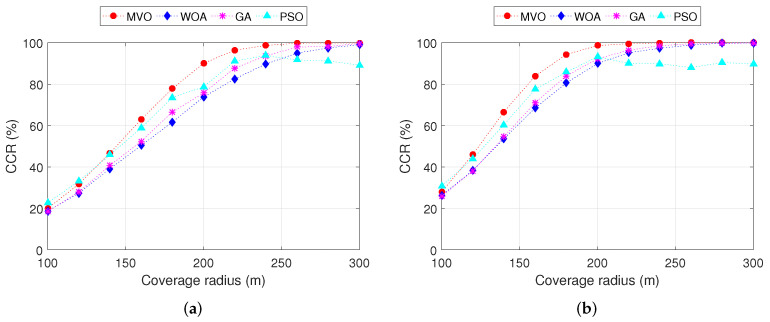
Performance comparison of the CCR versus the coverage radius of the routers. (**a**) INS-6. (**b**) INS-8.

Another metric also has a great influence on the performance of mesh routers placement algorithms in WMN; that is, the coverage radius of the mesh routers. This is more clearly visible in Figure 10, where we measure the CCR versus the coverage radius of the mesh routers for INS-6 and INS-8. We can observe that the wider the coverage area, the higher the CCR. This is obvious because the CCR is directly proportional to the width of the coverage radius of the mesh routers. Among the algorithms MVO, WOA, GA and PSO, MVO gives the highest efficiency for both INS-6 and INS-8. For INS-6 (Figure 10a), to be able to achieve 100% CCR, the required coverage radius for the MVO algorithm is 260 m. Meanwhile, this value for algorithms WOA, GA and PSO is greater than 300m. For INS-8 (Figure 10b), since the number of mesh routers in this instance is more than INS-6, the CCR is also better. The coverage radius of the mesh router only needs 220m, and the MVO algorithm can achieve a CCR of 100%. The details of the CCR values versus the coverage radius of the mesh routers for INS-6 and INS-8 are shown in Table 8.

### 4.6. Convergence Analysis of Algorithms

**Figure 11:**
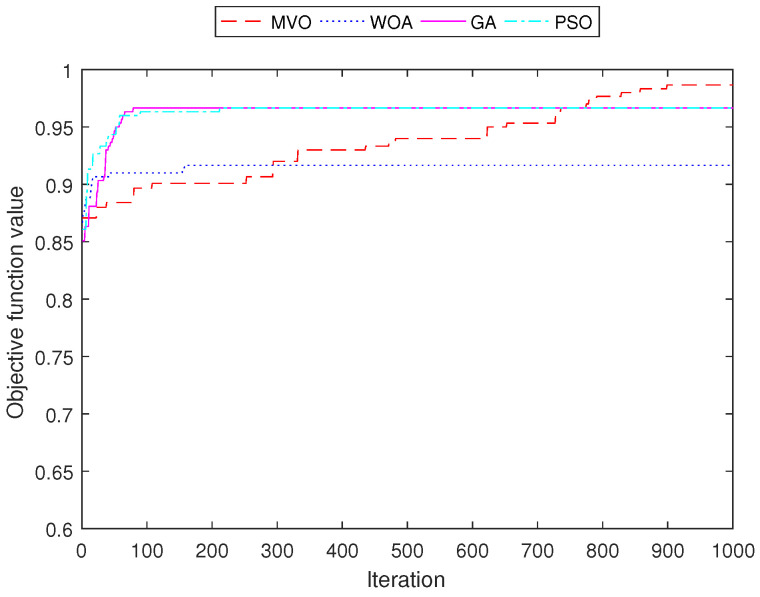
Performance comparison of the objective function value for INS-1 with 40 mesh routers.

**Figure 12:**
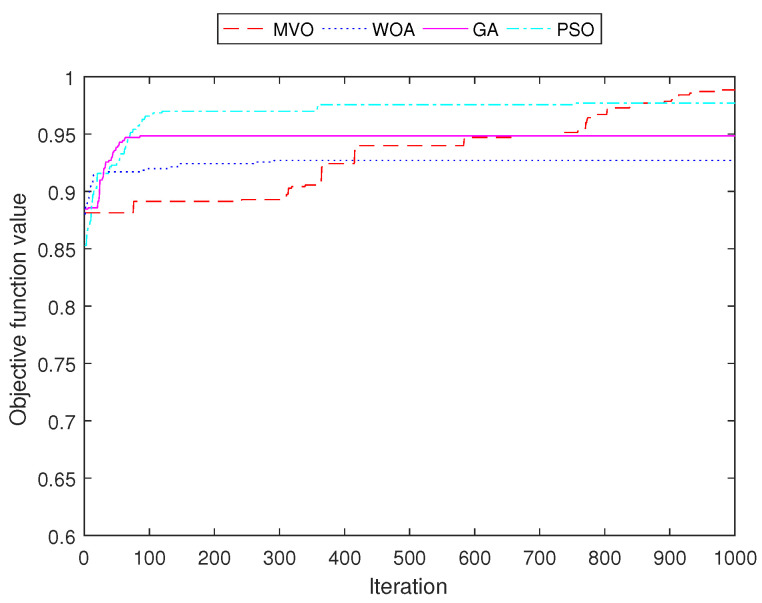
Performance comparison of the objective function value for INS-2 with 40 mesh routers.

**Figure 13:**
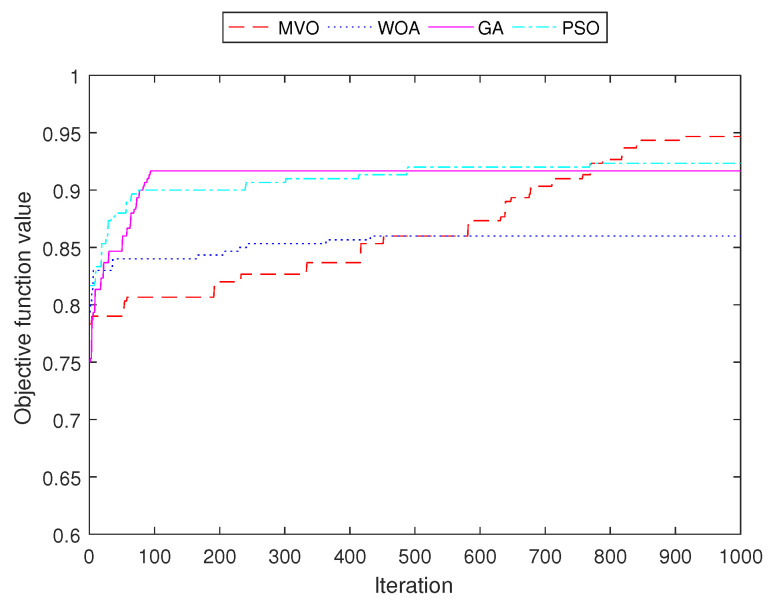
Performance comparison of the objective function value for INS-3 with 150 mesh clients.

**Figure 14:**
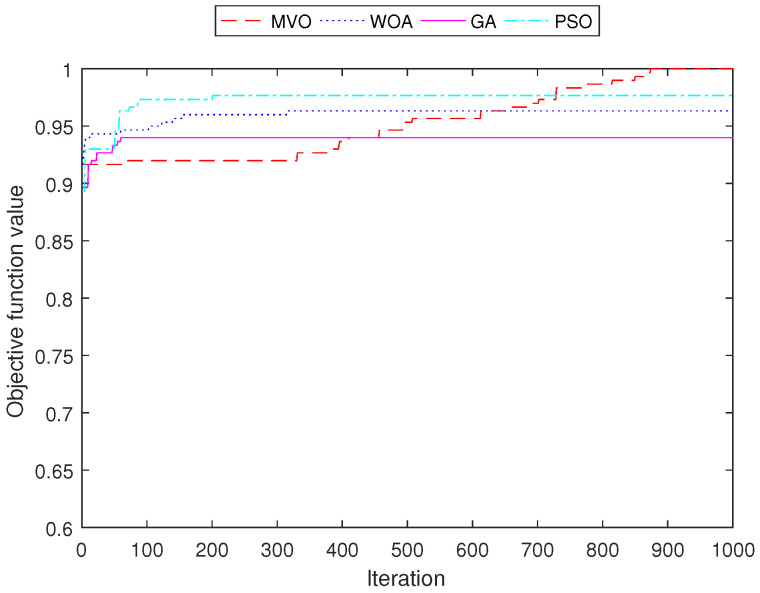
Performance comparison of the objective function value for INS-4 with 150 mesh clients.

Figure 11, Figure 12, Figure 13 and Figure 14 demonstrate the convergence curves of four algorithms for INS-1 with 40 mesh routers, INS-2 with 40 mesh routers, INS-3 with 150 mesh clients and INS-4 with 150 mesh clients, respectively. These convergence curves demonstrated that MVO is better than algorithms GA, PSO, and WOA. The algorithms GA, PSO, and WOA accrued early convergence. Although MVO has slow convergence, it is not stacked in local optima as with the other algorithms.

## 5. Conclusions

The MRP-WMN has attracted many research groups recently. Because this is an NP-hard problem, approximate optimization algorithms are typically used to solve it. We used the MVO optimization algorithm to solve the MRP-WMN in this paper. A new objective function for the MRP-WMN is also proposed, which takes two important performance metrics into account: connected client ratio and connected router ratio. The simulation method on Matlab is used to evaluate the performance of the MVO algorithm in solving the MRP-WMN problem. We ran simulations on a variety of network instances, changing the number of mesh routers, mesh clients, and coverage area. The simulation results show that the MVO algorithm outperforms the WOV, GA, and PSO algorithms in terms of connected client ratio and path loss.

In the next work, we will continue to develop algorithms by considering more constraints on quality of transmission and quality of service, such as signal-to-noise ratio (SNR), bit error rate (BER), and traffic load offered to each mesh router in order to improve the performance of WMN.

## Figures and Tables

**Table 1:** The notation used for formulating the node placement problem in the WMN.

Notation	Description
*m*	Number of mesh routers
*n*	Number of mesh clients
*k*	Number of gateway routers
$r_{i}$	The *i*-th mesh router
$R=\{r_{1},r_{2},\ldots ,r_{m}\}$	Set of mesh routers
$c_{i}$	The *i*-th mesh client
$C=\{c_{1},c_{2},\ldots ,c_{n}\}$	Set of mesh clients
$r_{gw_{i}}$	The *i*-th gateway router
$GW=\{r_{gw_{i}},r_{gw_{2}},\ldots ,r_{gw_{k}}\}$	Set of gateway routers
V=R∪C∪GW	Set of mesh nodes
*E*	Set of links between mesh nodes
G=(V,E)	Undirected graph topology describes WMN
Ψ(G)	Connected router ratio
Γ(G)	Connected client ratio
$d_{CR}$	Coverage radius of mesh routers
*W*	The width of the WMN area
*H*	The height of the WMN area
λ	Parameters control the metrics

**Table 2:** Settings of the MVO algorithm for the RNP-WMN problem.

MVO Algorithm	RNP-WMN Problem
Search space	WMN deployment area of dimensions W×H
Universe	Position of routers
Solution ($X^{best}$)	Set of optimal mesh routers locations
Inflation rate of universal	Objective function value

**Table 3:** The parameters of algorithms.

Algorithm	Parameter	Setting
MVO	Universes number	50
	WEP	Increase from 0.2 to 1
	TDR	Decrease from 0.6 to 0
WOA	Search-agent Number	50
	*a*	Decrease from 2 to 0
GA	Population size	50
	Crossover Rate	0.7
	Mutation Rate	0.01
PSO	Population size	50
	$c_{1}$	2
	$c_{2}$	2
	Inertia weight	1

**Table 4:** Simulation parameters.

Parameters	Setting
*n*	[100, 300] nodes
*m*	[10, 50] nodes
*k*	1 node
*W*	2000 m
*H*	2000 m
$d_{CR}$	[50, 200] m
λ	[0, 1]
Number of run	30
Number of iteration	1000

**Table 5:** Network instance use for simulation.

Instance	*m* (Routers)	*n* (Clients)	CR (m)
INS-1	[10, 45]	150	200
INS-2	[10, 45]	350	200
INS-3	30	[50, 400]	200
INS-4	45	[50, 400]	200
INS-5	30	150	[100, 300]
INS-6	30	350	[100, 300]
INS-7	45	150	[100, 300]
INS-8	45	350	[100, 300]

**Table 6:** Performance comparison of the number of connected clients and CCR when executing INS-1 and INS-2.

Instance	*n*	Number of Connected Clients				Connected Client Ratio (%)			
		MVO	WOA	GA	PSO	MVO	WOA	GA	PSO
INS-1	10	67.4	55.4	57.0	63.7	44.9	36.9	38.0	42.4
	15	91.7	74.0	75.2	87.7	61.1	49.3	50.2	58.5
	20	108.4	88.7	93.0	105.0	72.3	59.2	62.0	70.0
	25	124.0	103.3	108.4	116.3	82.7	68.9	72.3	77.5
	30	133.7	111.6	116.3	125.9	89.1	74.4	77.5	83.9
	35	140.1	120.2	129.5	131.8	93.4	80.1	86.3	87.9
	40	145.1	129.2	135.4	137.7	96.8	86.1	90.2	91.8
	45	147.5	134.0	139.4	139.2	98.4	89.3	93.0	92.8
INS-2	10	146.9	119.3	121.5	136.4	42.0	34.1	34.7	39.0
	15	200.9	157.9	163.5	185.9	57.4	45.1	46.7	53.1
	20	245.6	197.9	207.9	231.4	70.2	56.6	59.4	66.1
	25	285.9	229.8	240.4	261.0	81.7	65.6	68.7	74.6
	30	314.3	261.3	270.9	292.0	89.8	74.7	77.4	83.4
	35	330.5	279.4	290.3	310.2	94.4	79.8	83.0	88.6
	40	341.0	299.5	312.7	326.2	97.4	85.6	89.4	93.2
	45	344.9	314.9	325.5	329.9	98.5	90.0	93.0	94.3

**Table 7:** Performance comparison of the number of connected clients and CCR when executing INS-3 and INS-4.

Instance	*n*	Number of Connected Clients				Connected Client Ratio (%)			
		MVO	WOA	GA	PSO	MVO	WOA	GA	PSO
INS-3	50	46.2	40.3	41.9	42.6	92.3	80.6	83.9	85.2
	100	90.8	79.3	83.2	81.9	90.8	79.3	83.2	81.9
	150	132.5	113.1	122.0	125.9	88.3	75.4	81.3	83.9
	200	178.6	149.9	157.8	169.9	89.3	75.0	78.9	85.0
	250	220.7	181.0	190.5	206.7	88.3	72.4	76.2	82.7
	300	268.6	223.2	234.0	250.9	89.5	74.4	78.0	83.6
	350	314.9	253.4	263.2	292.8	90.0	72.4	75.2	83.6
	400	360.1	291.6	292.3	333.1	90.0	72.9	73.1	83.3
INS-4	50	49.8	47.4	48.6	47.4	99.7	94.7	97.2	94.8
	100	98.7	93.0	95.3	95.1	98.7	93.0	95.3	95.1
	150	146.5	134.9	136.0	138.6	97.7	89.9	90.7	92.4
	200	196.4	179.9	189.9	188.5	98.2	89.9	94.9	94.3
	250	246.0	221.3	233.8	230.4	98.4	88.5	93.5	92.1
	300	294.6	268.0	279.5	270.0	98.2	89.3	93.2	90.0
	350	343.9	311.9	325.3	315.1	98.2	89.1	92.9	90.0
	400	391.6	353.0	361.1	351.7	97.9	88.3	90.3	87.9

**Table 8:** Performance comparison of the number of connected clients and CCR when executing INS-6 and INS-8.

Instance	CR	Number of Connected Clients				Connected Client Ratio (%)			
	(m)	MVO	WOA	GA	PSO	MVO	WOA	GA	PSO
INS-6	100	70.0	65.4	64.6	79.2	20.0	18.7	18.5	22.6
	120	111.2	95.9	98.0	116.1	31.8	27.4	28.0	33.2
	140	163.6	137.2	142.7	160.7	46.7	39.2	40.8	45.9
	160	220.5	176.3	183.4	206.0	63.0	50.4	52.4	58.9
	180	272.5	216.0	233.0	257.5	77.9	61.7	66.6	73.6
	200	315.2	258.2	265.1	275.3	90.1	73.8	75.7	78.7
	220	337.5	289.0	307.0	319.0	96.4	82.6	87.7	91.1
	240	345.5	314.0	327.9	328.6	98.7	89.7	93.7	93.9
	260	349.3	332.6	342.8	321.0	99.8	95.0	97.9	91.7
	280	349.8	341.3	343.8	319.5	99.9	97.5	98.2	91.3
	300	349.8	346.8	348.6	312.1	99.9	99.1	99.6	89.2
INS-8	100	97.7	91.9	90.0	108.0	27.9	26.3	25.7	30.8
	120	160.9	134.2	133.6	153.7	46.0	38.3	38.2	43.9
	140	233.2	188.0	190.8	211.3	66.6	53.7	54.5	60.4
	160	293.7	239.6	248.7	271.2	83.9	68.4	71.1	77.5
	180	330.1	282.1	293.9	300.4	94.3	80.6	84.0	85.8
	200	345.3	314.9	322.8	326.1	98.6	90.0	92.2	93.2
	220	348.4	333.1	337.6	315.3	99.5	95.2	96.4	90.1
	240	349.8	340.8	345.1	314.5	100.0	97.4	98.6	89.9
	260	350.0	346.1	348.3	308.1	100.0	98.9	99.5	88.0
	280	350.0	349.3	348.9	316.5	100.0	99.8	99.7	90.4
	300	350.0	349.7	349.8	314.2	100.0	99.9	99.9	89.8

## Data Availability

Not applicable.
